# Photoelectron angular distributions as sensitive probes of surfactant layer structure at the liquid–vapor interface†

**DOI:** 10.1039/d1cp05621b

**Published:** 2022-02-03

**Authors:** Rémi Dupuy, Jakob Filser, Clemens Richter, Robert Seidel, Florian Trinter, Tillmann Buttersack, Christophe Nicolas, John Bozek, Uwe Hergenhahn, Harald Oberhofer, Bernd Winter, Karsten Reuter, Hendrik Bluhm

**Affiliations:** Fritz-Haber-Institut der Max-Planck-Gesellschaft Faradayweg 4-6 14195 Berlin Germany Reuter@fhi-berlin.mpg.de Bluhm@fhi-berlin.mpg.de; Chair for Theoretical Chemistry and Catalysis Research Center, Technische Universität München Lichtenbergstr. 4 85747 Garching Germany; Helmholtz-Zentrum Berlin für Materialien und Energie Albert-Einstein-Str. 15 12489 Berlin Germany; Institut für Kernphysik, Goethe-Universität Frankfurt am Main Max-von-Laue-Straße 1 60438 Frankfurt am Main Germany; Synchrotron SOLEIL, L'Orme des Merisiers, Saint-Aubin - BP 48 91192 Gif-sur-Yvette Cedex France; Chair for Theoretical Physics VII and Bavarian Center for Battery Technology, University of Bayreuth, Universitätsstraße 30 95447 Bayreuth Germany

## Abstract

The characterization of liquid–vapor interfaces at the molecular level is an important underpinning for a basic understanding of fundamental heterogeneous processes in many areas, such as atmospheric science. Here we use X-ray photoelectron spectroscopy to study the adsorption of a model surfactant, octanoic acid, at the water–gas interface. In particular, we examine the information contained in photoelectron angular distributions and show that information about the relative depth of molecules and functional groups within molecules can be obtained from these measurements. Focusing on the relative location of carboxylate (COO^−^) and carboxylic acid (COOH) groups at different solution pH, the former is found to be immersed deeper into the liquid–vapor interface, which is confirmed by classical molecular dynamics simulations. These results help establish photoelectron angular distributions as a sensitive tool for the characterization of molecules at the liquid–vapor interface.

## Abstract 2

Photoelectron angular distributions can yield information on the relative depth of different molecular groups at the liquid–vapor interface.

## Graphical abstract

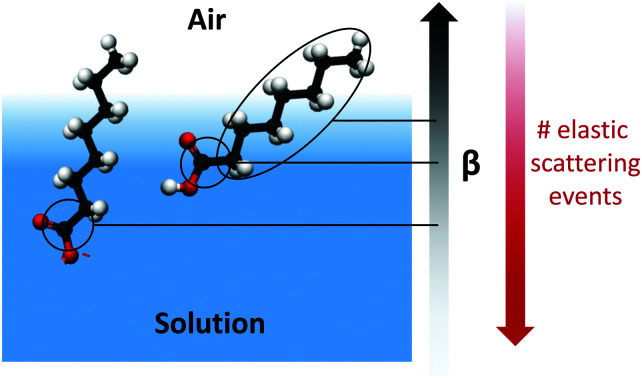

## 1 Introduction

The interface between aqueous solutions and air drives many important processes in the atmosphere and the environment.^1^ Of particular interest is the surface of liquid aerosols, whose properties remain poorly understood.^2^ Investigations of the chemical composition and the structure of these interfaces on well-controlled model systems using molecular-scale probes are necessary to gain further insights into their properties. The effects of organic surfactant molecules that accumulate at the surface of aqueous aerosols, and may significantly affect their physical and chemical properties,^3,4^ are among the chief questions regarding these systems.

X-ray photoelectron spectroscopy (XPS) has established itself as an important technique to study the liquid–vapor interface at the molecular level.^5,6^ In particular over the last 20 years XPS has been applied to the characterization of aqueous solutions, owing principally to the development of liquid microjet instruments^7^ but also to ambient pressure electron analyzers that enable operation at pressures in the mbar range, which are required when working on aqueous solutions in equilibrium with their vapor pressures. XPS primarily provides information on the chemical nature of solutes at the liquid–vapor interface, *e.g.*, the adsorption of organic molecules, or their protonation state.^8–10^ It also gives access to the electronic structure of the interface and the bulk of the solutions,^11–13^ which is important for the understanding of the details of (electro)chemical reactions.

XPS can also be used to obtain structural information about the depth distribution of species at the interface. The signal intensity in XPS depends, among other parameters, on the amount of losses by inelastic scattering experienced by photoelectrons between their point of emission and the detector, and thus on the depth at which they are located within the liquid layer below the liquid–vapor interface. Changing the photoelectron kinetic energy (KE), and thus their inelastic mean free path, by changing the incident photon energy then allows to perform depth-profiling of the solutes. This has been used in the past, *e.g.*, to determine the surface propensity of ions,^14–18^ or the orientation of molecules at the interface.^19,20^

One aspect of photoemission that has received less attention so far is the measurement of photoelectron angular distributions (PADs). In a PAD measurement the intensity of the photoelectron signal of a certain core level is monitored as a function of the angle between the linearly polarized electric field vector of the incident X-rays and the electron detection direction. In experiments on gas phase species, PADs depend on the interplay of the photon field with the molecular potential. In condensed phase experiments, however, additional factors come into play. In particular, elastic scattering will change electron trajectories, affecting the original PADs.^21,22^ PADs therefore reflect the degree of elastic scattering experienced by the emitted photoelectrons. This effect can be used to deduct information that is not present in a conventional XPS experiment that is performed at a fixed angle between the electric field vector of the incident X-rays and the electron detector axis.

PADs have been used previously to retrieve the inelastic mean free path of photoelectrons in pure water,^21^ an as of yet not precisely known quantity of great importance for the interpretation and quantification of XPS data of aqueous samples. But PADs can also be used for instance to obtain information on the relative depth at which the photoelectrons of the atoms of interest are generated. The similarity of PADs of a mixture of organosulfur compounds in aqueous solution has been used to infer that the depth distribution of the different species at the interface is likely very similar,^23^ although XPS spectra measured at a single detection angle would suggest otherwise. This application of PADs as a probe of the depth distribution of molecules at the interface is what we intend to explore further in this work. When trying to obtain information on the relative depth of different species at the interface, comparing XPS signal intensities (measured at a single detection angle) can be misleading or even impossible. The depth-profiling techniques mentioned above suffer from issues related to poorly constrained quantities such as the inelastic mean free path^24^ and photoionization cross-sections. PADs have the potential to be an important complementary tool here.

We focus in this work on solutions of a simple, atmospherically-relevant surfactant molecule, octanoic acid, which serves as a stand-in for generally abundant carboxylic acid molecules.^25^ Octanoic acid is a linear carboxylic acid, with an intermediate chain length between the short-chain, water-soluble and volatile acids, and the long-chain insoluble acids typically used in, *e.g.*, Langmuir film experiments. Using experimentally measured PADs and detailed molecular dynamics (MD) simulations, we aim to determine what information can be obtained from PADs on the arrangement of surfactant molecules at the interface. We investigate how PADs vary as a function of surfactant coverage and solution pH, shedding light for instance on the relative depth of protonated (octanoic acid) *vs.* deprotonated (octanoate) species. This kind of information is relevant not only for a fundamental understanding of the properties of surfactants at liquid–vapor interfaces, but also for the role that the surface propensity of surfactant and solute species plays in heterogeneous reactions with gas-phase species. It helps establishing PAD measurements as a useful tool in the characterization of liquid–vapor interfaces with XPS.

## 2 Methods

### 2.1 XPS experimental setup

XPS experiments were performed using the SOL^3^PES setup^26^ at the UE52_SGM beamline of the BESSY II synchrotron facility. A liquid jet with a diameter of 30 μm and a velocity of ∼20 m s^−1^ was used inside a vacuum chamber with a base pressure of around 3 × 10^−4^ mbar during the jet experiments. After traversing the vacuum chamber the liquid jet was frozen out using a cold trap (a metal cylinder filled with liquid nitrogen). The hemispherical electron energy analyzer aperture (∼500 μm diameter) was at a distance of ∼0.5 mm from the jet, at a detection angle orthogonal to the jet axis. The incident X-ray beam from the beamline was focused into a spot of approximately 450 μm onto the liquid jet. The propagation direction of the beam was orthogonal to both the liquid jet axis and the detection axis. The direction of the linear polarization at the UE52_SGM beamline is tuneable and was varied from 0° to 90° relative to the detection axis for the measurement of photoelectron angular distributions.

Photoelectron spectra were taken mostly in the C 1s region at 450 eV photon energy (*hν*), *i.e.*, ∼155 eV photoelectron KE. This corresponds to a probing depth of approximately 1.5 nm.^21^ The binding energies (BE) indicated are obtained simply from BE = *hν* − KE, without further calibration, as the absolute binding energies are of no interest in the analysis for the present investigation.

For calibration and normalization purposes (see below) additional gas-phase PAD experiments were performed at the PLEIADES beamline of the SOLEIL synchrotron. These experiments were conducted with the liquid jet setup of the beamline (see *e.g.* ref. 27) installed. The liquid jet was not running and instead gas-phase pentanoic acid was injected from the vapor above a bulk pentanoic acid sample through the catcher aperture of the liquid jet setup, mimicking the conditions in which liquid jet experiments are performed on this beamline. Pentanoic acid was used instead of octanoic acid because of its higher vapor pressure. We have previously confirmed that the PAD results obtained on similar solutions were the same for the liquid jet setups on the UE52_SGM and PLEIADES beamlines.

The solutions were prepared using octanoic acid and sodium octanoate (>99%, Sigma-Aldrich) as purchased and MilliQ water with 50 mM NaCl added to ensure sufficient conductivity of the solutions, a prerequisite for liquid jet photoemission experiments. The pH of the solutions was adjusted using concentrated HCl or NaOH.

### 2.2 XPS data analysis

Photoelectron spectra were fitted using the KolXPD software. A linear background and Gaussian profiles were used for all peaks. Some peaks show asymmetries, as will be discussed later in the text, and were therefore fitted with multiple Gaussians. For series of PAD measurements, all spectra of the same series are fitted using the same parameters for the FWHMs and at fixed relative positions of the peaks. A linear offset of the BE axis (which affects all peak positions equally) was allowed to account for slight differences between spectra due to, *e.g.*, variations in the streaming potential of the jet.^13^ For the liquid data acquired at UE52_SGM, the spectra were normalized to the mirror current measured on the last mirror of the beamline optics, so as to account for slight differences in photon flux at different polarization angles. For the gas-phase data acquired at PLEIADES, a similar flux correction was made by measuring the photon flux on a photodiode.

As will be shown below, the C 1s spectra show distinct peaks for the CH_*x*_ and COOH (COO^−^) species of octanoic acid (octanoate). The PAD for each of these species was calculated from their integrated peak intensities as a function of polarization direction using the following formula:1
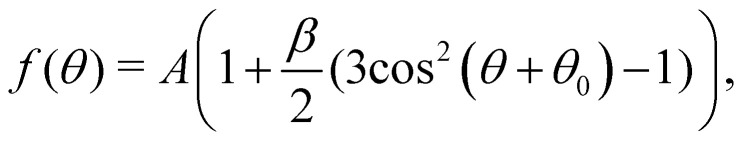
where *θ* is the angle between the polarizarion direction and the detection axis, and *A*, *β*, and *θ*_0_ are fit parameters. *β* is the physically relevant asymmetry parameter that describes the PAD, while *A* and *θ*_0_ are only adjustment parameters. *θ*_0_ allows to account for a possible slight difference between the nominal and actual polarization direction, which is a systematic error introduced by, *e.g.*, a slight misalignment of the elliptically polarized undulator. We consistently found a value of *θ*_0_ ≈ 10° in all fits of the data collected at UE52_SGM. *A* is a scaling factor.

This formula assumes a degree of linear polarization *p* = 1. This is not likely to be completely true, but would only change the absolute value of *β* and not the relative ones, as *β* and *p* are almost degenerate parameters when *p* is included in the fit formula.^28^ Since there is no easy way to determine the degree of polarization of the synchrotron radiation beam used here, we stayed with the assumption that *p* = 1. Similarly, we neglected the effect of the finite angular acceptance of the analyzer, which is not well-known in the configuration used here.

The above formula is derived for linearly polarized incident light in the case of randomly oriented molecules,^29^ and is strictly valid only in this case. One could question whether molecules really are randomly oriented in the present case, as it can be expected that long-chain surfactant molecules will tend to orient themselves with the hydrophobic tail pointing towards vacuum. Sampling over part of the circular jet certainly randomizes the orientation distribution of the molecules to some degree, although not completely. The fits we obtain with this formula are nonetheless very good, which suggests that the preferential orientation of the molecules does not influence our results.

The relative uncertainties of the *β* values extracted from the fits depend on many factors such as noise in the spectra, photon flux variability, jet fluctuations, and choices made when fitting the spectra. Considering the sensitivity of the PAD fits to small changes of intensity, we estimate that differences of *β* values lower than 0.02 cannot be trusted. Actual error bars for individual values are however difficult to estimate.

### 2.3 Surface tension measurements

Surface tension measurements of octanoic acid and sodium octanoate solutions of varied bulk concentrations were performed in order to characterize the surface density of molecules for the solutions used in the XPS experiments. Surface tension was measured using the EZ-Pi tensiometer instrument from Kibron Inc, Helsinki. Measurements are based on the Wilhelmy method^30^ and a freshly flamed Platinum probe was used to ensure its full wetting by the liquid.

### 2.4 Molecular dynamics simulations

Complementary to the above described experiments, classical MD simulations are employed to investigate the behavior of an octanoic acid surfactant on a water surface. Here we consider the high and low pH limits by assuming that all acid molecules are either deprotonated or protonated in these limits, respectively.

The MD simulations were conducted using the LAMMPS^31,32^ package, version 24 Dec 2020. The SPC/Fw^33^ model for water was used. OPLS-AA force fields with (1.14*)CM1A charges for octanoic acid and octanoate were generated using the LigParGen^34–36^ tool. The charges, which to some degree depend on the model geometry, are reported explicitly in the ESI† for the sake of reproducibility. It is worth noting that actual (de)protonation reactions lie outside the scope of this model, which is based on a fixed bond environment. Despite this limitation, OPLS-AA has been used in other studies to investigate the behaviour of octanoic acid^37^ as well as octanoate^38^ in or on the surface of aqueous solutions, in conjunction with other versions^39,40^ of the SPC family of water models. For sodium cations, we used a Lennard-Jones (LJ) potential with *ε* = 0.01814 kcal mol^−1^ and *σ* = 3.206 Å.^41^ This has been shown to correctly reproduce chemical potentials and specific volumes in the low concentration range in which we are interested, while also being compatible with the geometric mixing rule for *σ* of OPLS-AA.^42^

The water box was generated using fftool^43^ and packmol,^44^ version 20.2.2. The water–air interface was modeled as a water slab surrounded by vacuum. For all simulations, a 50 Å × 50 Å × 50 Å water box containing 4179 water molecules was used for the initial geometry, which amounts to a density of approximately 1 kg L^−1^. The simulation cell was chosen to extend 200 Å in *z* direction, with 100 Å of vacuum on top of the initial water box and 50 Å below. LJ walls were placed 5 Å above the lower and below the upper cell boundary to prevent molecules leaving the simulation cell. Periodic boundary conditions were applied in *x* and *y* direction. In *z* direction, periodic boundary conditions were formally applied, but non-periodicity was mimicked by inserting empty volume between the slabs equivalent to three times the height of the cell and removing inter-slab dipole interactions.^32^

The MD simulations were conducted in the *NVT* ensemble by applying a Nosé–Hoover thermostat^45,46^ with fixed cell dimensions at 300 K. Drift of the water box as a whole in *z* direction was prevented by subtracting the center of mass velocity from the velocities of all atoms in each time step and rescaling the velocities to conserve kinetic energy.^32^ Time steps of 1 fs were used. All systems were equilibrated for 1 000 000 time steps before running 10 000 000 time steps of production calculation. For evaluation, every 1000th MD step of the latter was used as a snapshot.

**Fig. 1:**
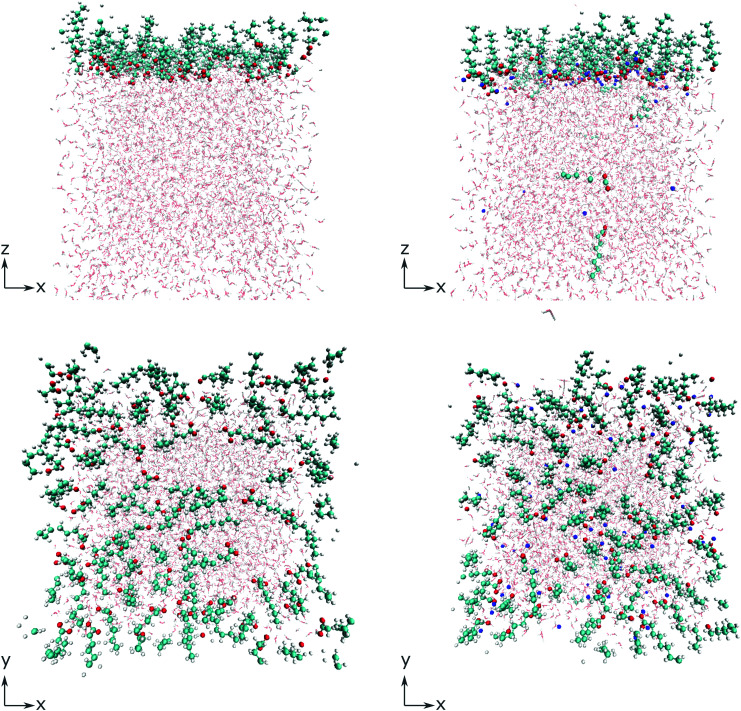
Simulation cells with 64 surfactant molecules per cell immediately after equilibration, for octanoic acid (left) and the octanoate anion with a Na^+^ counterion (right), in side view along the *y* axis (top) and top view along the *z* axis (bottom). C: turquoise spheres, H: small white spheres, O: red spheres, Na: blue spheres. Water molecules depicted in lighter colors.

To gain deeper insight into the adsorption mechanism at different coverages, simulations for both the octanoid acid and the octanoate anion with Na^+^ as counterion were run with coverages of 2^*n*^ molecules per simulation box in a range from *n* = 1…256 molecules, corresponding to coverages of 4.0 × 10^12^ cm^−2^ to 1.024 × 10^15^ cm^−2^. All molecules and ions were initially placed above the water slab. The geometries of the simulation cells with 64 molecules per cell, immediately after equilibration, are shown in Fig. 1 as an example.

We are interested in the time averaged vertical positions of the surfactant's C atoms relative to the water surface. To define the spatially averaged location of the water surface (or, more precisely, the surface of the polar phase) in a snapshot, a density function2
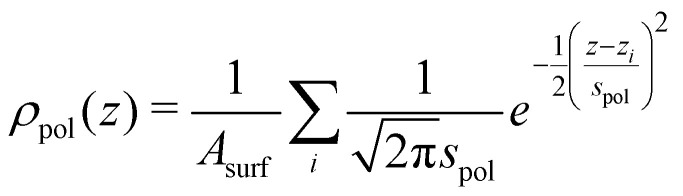
with a broadening *s*_pol_ = 1 Å, the surface area of the simulation cell *A*_surf_ = 2500 Å^2^, and *z*_*i*_ the *z* coordinates of all O atoms (both of water and the carboxylic acid/carboxylate) as well as Na^+^ ions was used. In particular in the high coverage situation, the surfactant molecules and their counterions constitute a significant fraction of the overall polar medium, which is why they also need to be considered in the density function. The water surface in each snapshot was defined as the *z* value at which *ρ*_pol_ had decayed to 0.015 Å^−2^, which is approximately half the average bulk value.

The vertical positions of the adsorbed molecules’ C atoms were determined relative to this average water surface. To quantify an average vertical distribution of the *j*-th C atom in the chain, we employed a similar density function,3
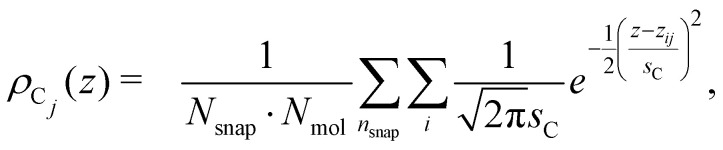
where *z*_*ij*_ is the *z* coordinate of the *j*-th C atom in the chain of the *i*-th octanoic acid/octanoate molecule, relative to the spatially averaged water surface in the respective snapshot. Here, a Gaussian broadening of *s*_C_ = 0.5 Å was employed and *ρ*_C*j*_ was normalized by the number of MD snapshots *N*_snap_= 10 000 and the number of octanoic acid/octanoate molecules *N*_mol_ in the system. For the case of the anion, an average vertical distribution of the Na^+^ counterions was defined in complete analogy.

In addition to the vertical position of the C atoms, the 2D pair correlation function of the carboxylic C atoms was considered. Its histogram form, normalized by the total number of pairs per surface area, is given by4
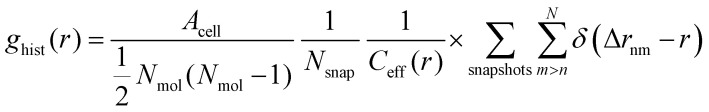
with the *x*,*y* cross section area of the cell *A*_cell_ and the effective circumference *C*_eff_(*r*) of a circle of radius *r* cut at the edge of the unit cell. *r* is sampled in steps of Δ*r* starting at ½Δ*r* and *δ*(Δ*r*_nm_−*r*) = 1 if −½Δ*r* ≤ Δ*r*_nm_ – *r* < ½Δ*r* and 0 otherwise. A step size of 
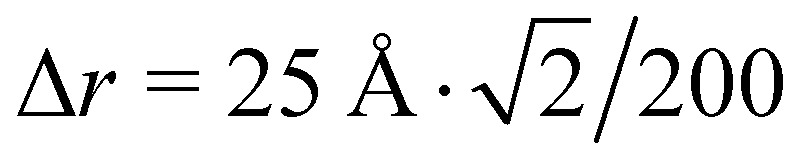
 is used. Δ*r*_nm_ is the distance between C atoms *n* and *m*, wrapped to the first unit cell (centered around either of the atoms). It is calculated only in the *x*,*y* plane, in consistency with the assumption of a flat water surface, as5
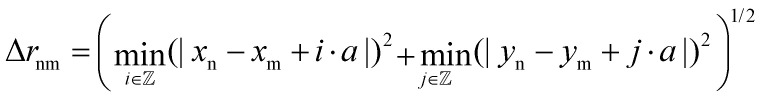
with cell size *a*. To reduce noise, the actual pair correlation function *g*(*r*) is then approximated as the convolution of *g*_hist_(*r*), mirrored at the origin, with a Gaussian distribution. This is mostly consistent with the evaluation of the vertical distributions, with only two differences. First, to account for the more fine grained structure of *g*(*r*), a broadening of *s* = 0.25 Å is used and second, the set of Δ*r*_nm_ is first discretized into bins of width Δ*r*, instead of evaluating the Gaussians around the exact positions. This is necessary to improve computational efficiency since the number of pairs nm increases quadratically with the number of molecules.

## 3 Experimental results

### 3.1 Surface adsorption of octanoic acid and sodium octanoate

The surface density of surfactants is an important parameter, and we therefore performed experiments to estimate how the bulk concentration of octanoic acid and sodium octanoate relate to their surface density. For octanoic acid, the maximum concentration of 4 mM used in our experiments is close to the solubility limit. For sodium octanoate, higher concentrations are in principle possible but were avoided because they approach the critical micelle concentration.^47^ The surface density can be determined from surface tension measurements.

**Fig. 2:**
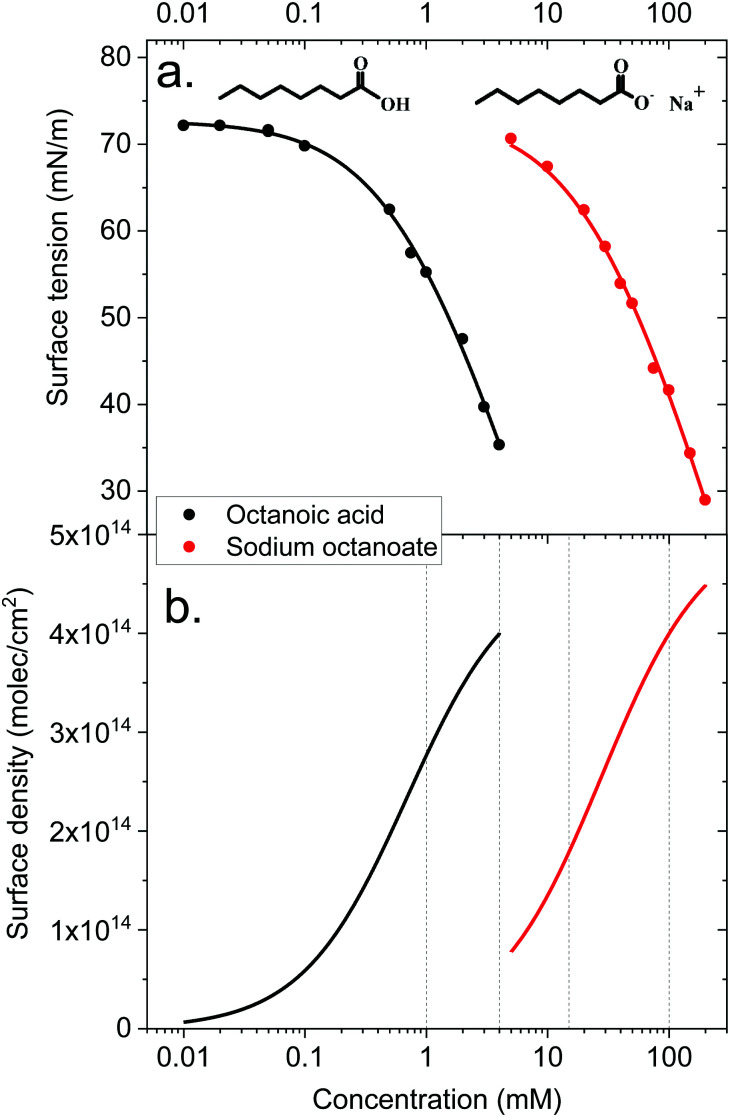
Top panel: Surface tension of octanoic acid (black dots) and sodium octanoate (red dots) solutions, as a function of solute concentration. The experimental data is fitted with the Szyszkowsky equation as detailed in the text. Bottom panel: Langmuir adsorption curves (surface density of molecules as a function of bulk solute concentration) derived from the surface tension data. The derivation is described in the text.

Fig. 2a shows the measured surface tension of solutions of octanoic acid (pH = 3) and sodium octanoate (pH = 9) as a function of solute concentration. These data are well fitted by the Szyszkowski equation, which is derived from a combination of the Gibbs adsorption equation and the Langmuir isotherm model,^48^6
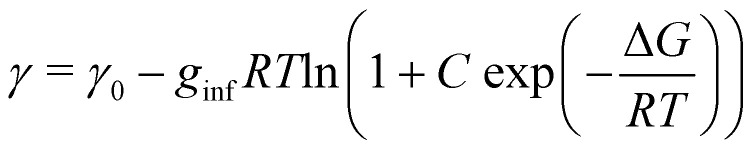
where *γ*_0_ is the surface tension of pure water, *g*_inf_ a scaling term, *C* is the bulk solute concentration and Δ*G* the free energy of adsorption of the solute at the surface. We obtain Δ*G* = −27.7 kJ mol^−1^ for octanoic acid, and Δ*G* = −18.6 kJ mol^−1^ for sodium octanoate, which as expected highlights the higher surface propensity of the protonated form.

The surface density of molecules can be estimated from the derivative of the surface tension curve, through the Gibbs adsorption equation:7
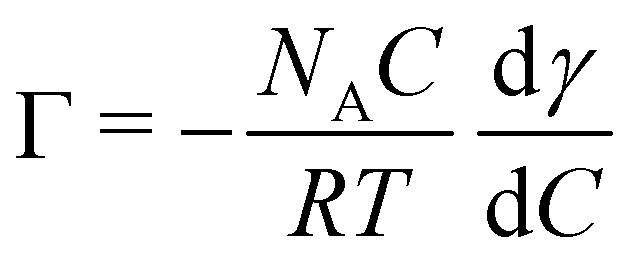
where *Γ* is the surface excess or surface density of molecules (in molecules per unit of area) and *N*_A_ the Avogadro constant. The surface excess curves obtained from applying this equation to the fitted surface tension curves are displayed in Fig. 2b. The vertical dashed lines indicate the bulk concentrations of the solutions used in the XPS experiments presented below. We can estimate, from experiments performed on long-chain linear carboxylic acids (*e.g.*, stearic acid) films on water, that the surface density of a closely packed monolayer is ∼5 × 10^14^ molec cm^−2^. Extrapolating this value to the medium-sized octanoic acid, the value of 4 × 10^14^ molec cm^−2^ estimated at the highest bulk concentrations used would correspond to a surface coverage of roughly 0.8.

These surface tension measurements offer a first limited information on carboxylic acid behaviour at the liquid–vapor interface. However, no microscopic information on the orientation of molecules, their location relative to the interface and so on can be derived from these macroscopic measurements. This is why we turn to MD simulations and photoemission spectroscopy to obtain a more detailed picture.

### 3.2 Gas-phase XPS spectra and photoelectron angular distributions

**Fig. 3:**
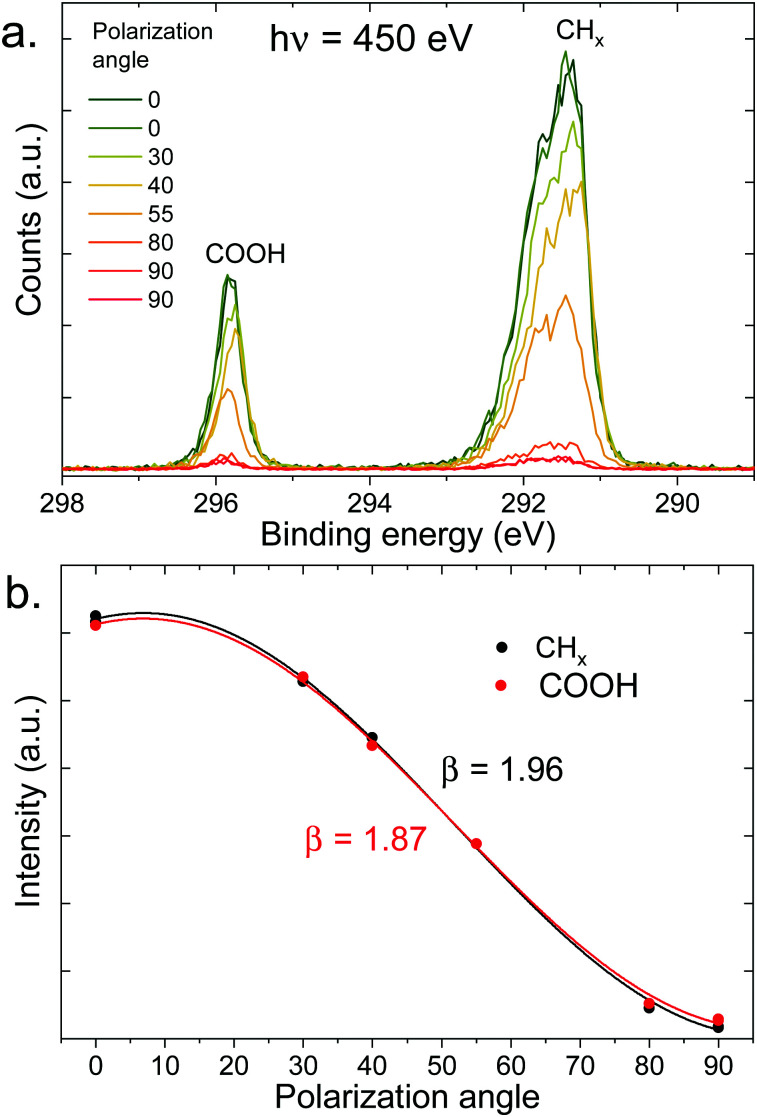
(a) C 1s photoelectron spectra (450 eV) of gas-phase pentanoic acid, at different polarization angles of the incident beam (angle relative to the detection direction). The extreme angles (0° and 90°) have been measured twice here. (b) Integrated intensity of the peaks of the top panel (normalized at 55°) as a function of polarization angle, fitted with eqn (1) to extract the value of *β* indicated in the figure.

As a reference and normalization for the measurements in solution, gas-phase spectra of a linear carboxylic acid were measured at a photon energy of 450 eV. This corresponds to a C 1s photoelectron kinetic energy of ∼160 eV. As the vapor pressure of octanoic acid (∼10^−3^ mbar) is too low for these measurements, we used pentanoic acid (vapor pressure of ∼1 mbar) as a proxy. The spectra at different polarization angles are displayed in Fig. 3a and the resulting PADs in Fig. 3b. The peaks labeled CH_*x*_ (hydrocarbon tail) and COOH were fitted with 3 and 2 Gaussian components, respectively, to account for the vibrational progression giving rise to the pronounced asymmetry of the peaks.^49^ This vibrational progression is not well resolved here as moderate spectral resolution settings were used.

As seen in Fig. 3b, pentanoic acid already exhibits a difference in the anisotropy parameter *β* between the COOH group and the CH_*x*_ hydrocarbon tail. Both values of *β* deviate from the ideal value of 2, although the value for CH_*x*_ is very close considering that our experiment necessarily has a non-zero solid angle of detection. The value for COOH on the other hand clearly deviates from 2. The ratio between the area of the two peaks, evaluated at a 55° (*i.e.*, “magic”) angle where angular effects cancel out, is 4.4 ± 0.1, and therefore also deviates from the expected stoichiometric value of 4. These differences in anisotropy and total cross-section between the CH_*x*_ and COOH group at 450 eV (*i.e.*, 160 eV photoelectron KE) can be attributed to intramolecular scattering of the outgoing photoelectron wave. Oscillations in the total absorption cross-section of different carbon functional groups as a function of photon energy have already been observed in the gas phase and in solution previously, using heavy chlorine atoms bound to the carbon to emphasize scattering.^50,51^ The results shown here demonstrate that such effects can be observed even with more common oxygen-bound carbon groups. Additionally, the non-stoichiometric value observed for the peak ratios could also originate (at least in part) from a loss of intensity of the COOH peak due to shake-up π–π* transitions. The conclusion from these measurements is that caution is required when interpreting peak ratios and PADs for molecules in solution, if a gas-phase reference is not available.

### 3.3 XPS and photoelectron angular distributions of the aqueous solutions

**Fig. 4:**
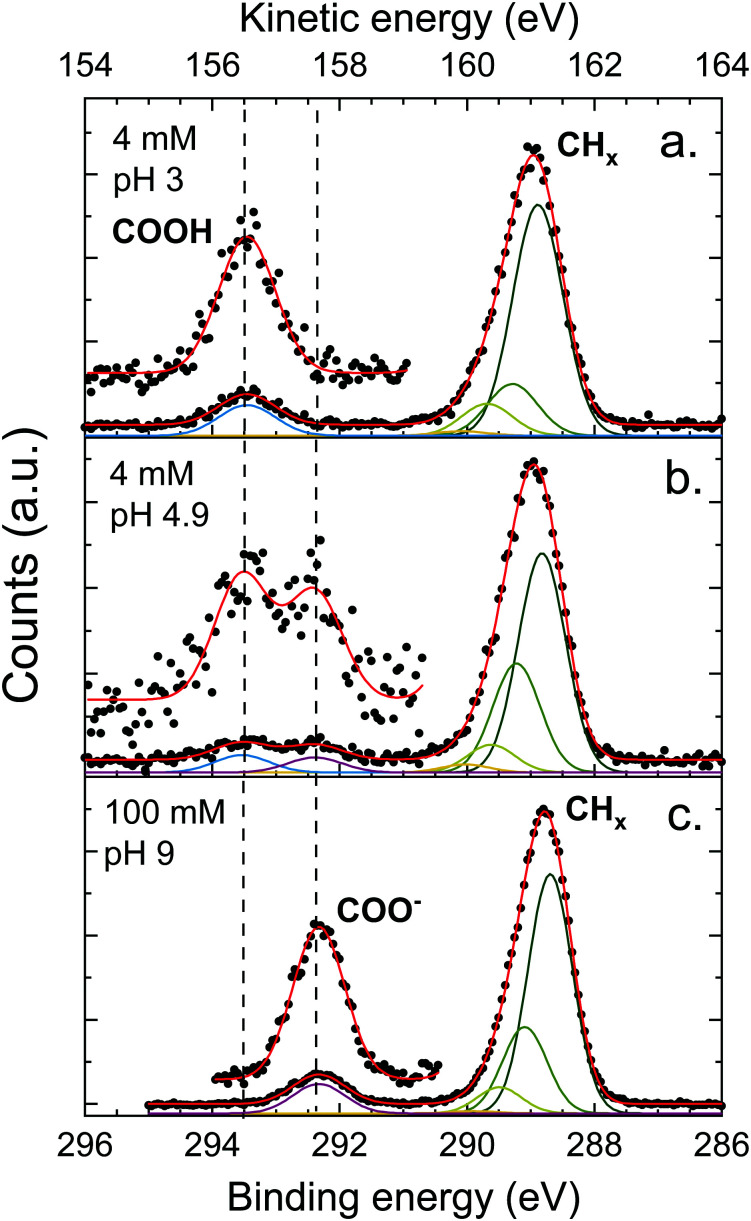
C 1s photoelectron spectra of octanoic acid solutions at different pH, measured at the magic angle (55°). The assignment of the different peaks is specified in the figure and dashed lines mark the position of features further discussed in the text. Several Gaussians were used to fit the peak attributed to CH_*x*_ (hydrocarbon tail) to account for the asymmetry of the peak, as discussed in the text. A blow-up of the high-energy part of the spectrum is also displayed to show the peaks more clearly.

The XPS spectra of octanoic acid solutions at different pH values and concentrations in the C 1s region are shown in Fig. 4. The upper panel shows the data for a solution at pH 3, where only the protonated form of the octanoic acid is present in solution. The peak at 289 eV is attributed to the hydrocarbon chain carbons CH_*x*_, while the one at 293.5 eV is attributed to the COOH group carbon. The bottom panel shows the spectrum of a 100 mM solution at pH 9, where the acid is entirely deprotonated and thus the solution is purely sodium octanoate. Again a CH_*x*_ peak is observed at 288.8 eV, along with a peak at 292.3 eV attributed to the COO^−^ group carbon. The protonated and deprotonated forms of octanoic acid are thus easily distinguished in XPS by a shift of more than 1 eV between the C 1s peaks of the functional group carbon. In the middle panel, the spectrum for a solution at pH 4.9, which corresponds to the bulk p*K*_a_ of octanoic acid, is shown. Here both COOH and COO^−^ peaks are observed, with a roughly equal intensity. This suggests that the apparent surface p*K*_a_ of octanoic acid is the same as its bulk p*K*_a_, something already measured in surface tension experiments.^52^ This is a surprising result considering the large differences in surface propensity of octanoic acid and octanoate, but we will not explore it further in the present work.

The CH_*x*_ peaks in solution still display an asymmetry, although it is less pronounced than in the gas phase. We accordingly attempted to fit this peak with a vibrational progression. However, the fit obtained for the pH 3 solution (octanoic acid) does not resemble a vibrational progression, and the width and asymmetry of the CH_*x*_ peaks are quite different for different pH values. This suggests that additional effects come into play and the fit of the vibrational progression does not hold physical meaning. These effects could include a second-neighbour effect on the carbon closest to the COO group, or different hydration structures. As this is of little importance for the analysis of the PADs we do not analyse this asymmetry further here. The COOH and COO^−^ peaks are well fitted by a single Gaussian.

The ratio between the CH_*x*_ and COO^−^ or COOH peak signals differ for the different solutions, and also from the ratio obtained for the gas phase (4.4 for pentanoic acid, *i.e.*, 1.1 per CH_*x*_ carbon atom). The expected stoichiometric ratio for octanoic acid is 7. The observed ratio, however, is higher: 8.9 for a 4 mM solution at pH 3, 10.4 for 100 mM at pH 9, and 9.0 for 4 mM at pH 4.9, which amounts to 1.3–1.4 per CH_*x*_ carbon atom, higher than in the gas phase. This behavior has already been observed multiple times for other carboxylic acids, as well as alcohols,^20,53^ yielding very similar ratios. It will be discussed further below.

**Fig. 5:**
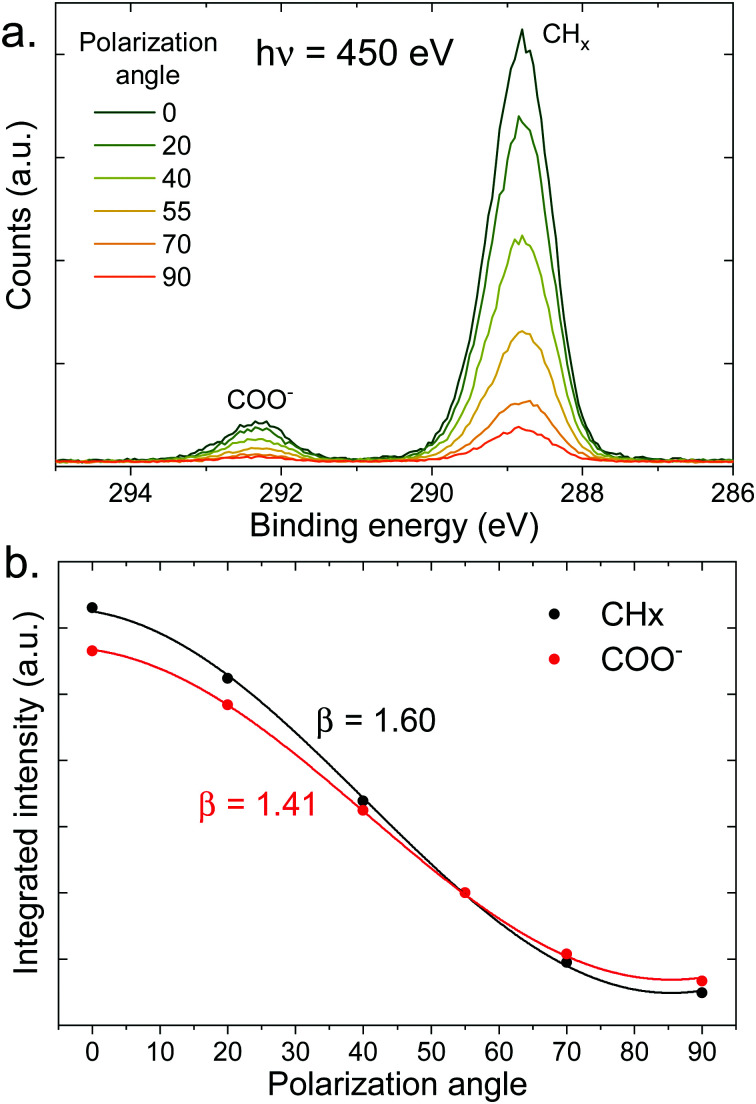
(a) C 1s photoelectron spectra (450 eV) of a 100 mM pH 9 solution of sodium octanoate, at different polarization angles of the incident beam (angle relative to the detection direction). (b) Integrated intensity of the peaks of the top panel (normalized at 55°) as a function of polarization angle, fitted with eqn (1) to extract the value of *β* indicated in the figure.

Let us now move on to the PAD measurements. In the top panel of Fig. 5, a series of C 1s spectra at different polarization angles is shown for the 100 mM sodium octanoate solution at pH 9. The intensity of each peak is integrated and fitted as a function of polarization angle (see bottom panel of Fig. 5). The *β* values obtained from the fits are indicated in the figure. The same procedure was repeated for a number of different solutions with varying pH and concentration.

In order to isolate liquid-phase differences in the PADs, we first need to correct for the differences already present in the gas phase. We thus introduced a value *β*′ normalized to the gas-phase value: 
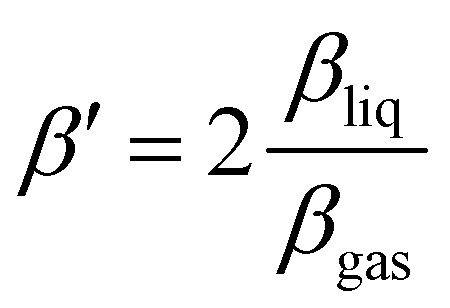
. *β*′ is therefore the value that *β*_liq_ would have if *β*_gas_ was equal to 2, the value expected for a 1s orbital without any scattering. The *β*′ values extracted from the fits are summarized in Table 1, along with the CH_*x*_ to COO (sum of COOH and COO^−^) ratios. We assumed that the gas-phase value for COOH and COO^−^ is the same. The ratios have been normalized to the ratio per CH_*x*_ carbon atom in the gas phase (here 1.1). A similar table containing the uncorrected values can be found in the ESI.†

**Table 1:** Summary of the ratio of CH_*x*_ to COO intensity (at the magic angle) and angular distribution parameters for gas-phase pentanoic acid and the different solutions. For gas phase pentanoic acid (first column) we report the *β* parameters, while for solutions we report *β*′, which is the *β* parameter normalized to the gas phase (see text). The ratios in solution have also been normalized with respect to the gas-phase values. Uncorrected values are given in the ESI

	Pe. acid	Oc. acid (pH 3)		Oc. acid/octanoate		Na octanoate (pH 9)	
	Gas	1 mM	4 mM	4 mM pH 4.6	4 mM pH 4.9	15 mM	100 mM
Surf. dens. (molec cm^−2^)		2.8 × 10^14^	4 × 10^14^			1.8 × 10^14^	4 × 10^14^
CH_*x*_	1.96	1.72	1.67	1.71	1.72	1.66	1.63
COOH	1.87	1.68	1.65	1.67	1.68		
COO^−^				1.57	1.59	1.53	1.51
CH_*x*_/COO ratio	4.4	7.3	8.1	8.0	8.2	8.0	9.5

The following observations can be made:

• The value of *β*′ for the CH_*x*_ peak is systematically higher than the value for the COOH or COO^−^ peak. The values for CH_*x*_ and COOH are, however, very close to each other, sometimes with differences below what we estimate can be considered as a significant effect (±0.02).

• At a given pH, increasing the concentration of the solution leads to a reduction of the values of *β*′ for all peaks.

• The *β*′ value for COO^−^ is systematically lower than that of COOH, including the case where both protonated and deprotonated species coexist at the surface (pH = 4.9).

The observation of a consistently lower *β*′ value for COO^−^ compared to COOH points to stronger elastic scattering for photoelectrons emanating from COO^−^ and is thus indicative of a deeper penetration of the COO^−^ species into the solution compared to the COOH species. In the next section MD simulations of octanoic acid and sodium octanoate will be discussed, which support this interpretation.

## 4 Simulation results

### 4.1 Acid

The adsorption behavior of octanoic acid can be roughly categorized into three regimes: ‘Low coverage’, ‘≲1 ML coverage’, and ‘high coverage’. The transition from low to ≲1 ML coverage occurs around 3.2 × 10^13^ cm^−2^, corresponding to 8 molecules in our simulation cell. The transition from ≲1 ML to high coverage occurs somewhere between 2.56 × 10^14^ cm^−2^ (64 molecules per simulation cell) and 5.12 × 10^14^ cm^−2^ (128 molecules per simulation cell). This is in agreement with experiment, which finds a coverage of 4 × 10^14^ cm^−2^ for a nearly saturated solution of octanoic acid, *cf.* Section 3.1.

In the low coverage regime, no pronounced coverage-dependent trends are observed. The mean *z* positions of the C atoms relative to the water surface exhibit only minuscule changes with coverage, see Fig. 7a. On average, the alkyl chains lie relatively flat on the water surface, with the vertical distributions being wider for C atoms at the methyl end of the alkyl chain, *cf.*Fig. 9a. This behavior may be due to thermal motion, minimization of the surface tension of water, or a combination of both. A clear maximum of the pair correlation function of the carboxylic C atoms can be observed in the first coordination shell around 5 Å, *cf.*Fig. 8a, indicating an attractive interaction between the acid molecules.

**Fig. 6:**
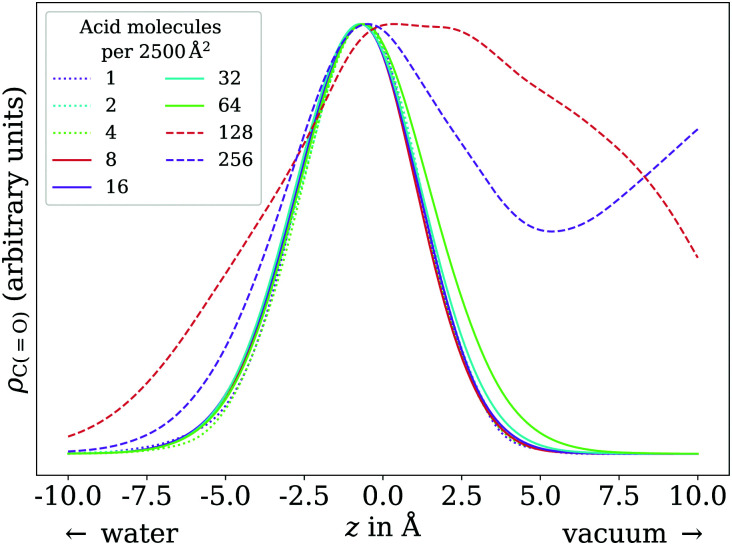
Vertical distributions of the carboxylic C atom of octanoic acid in the low (dotted lines), ≲1 ML (solid lines), and high coverage (dashed lines) regimes, normalized by the respective maximum value for better comparability. High coverage distributions on a wider *z* range shown in the ESI.†

**Fig. 7:**
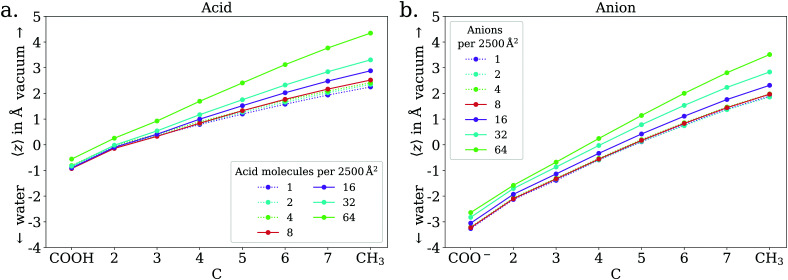
Mean vertical positions of all C atoms relative to the spatially averaged water surface in the low (dotted lines) and ≲1 ML coverage (solid lines) regimes, for octanoic acid (a) and octanoate (b).

**Fig. 8:**
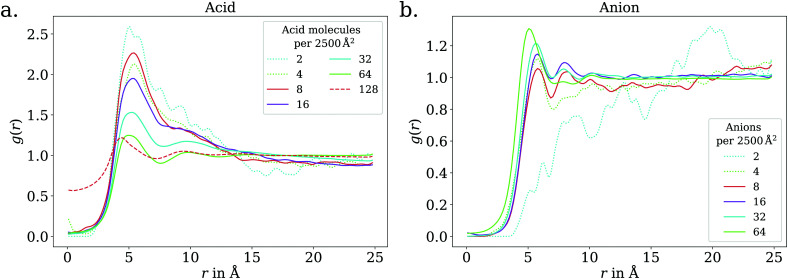
2D pair correlation function of the carboxylic C atoms in the low (dotted lines) and ≲1 ML coverage (solid lines) regimes, for octanoic acid (a) and octanoate (b). Additionally, for octanoic acid, data for 128 molecules per unit cell, *i.e.*, >1 ML, is shown in dashed lines. At even higher coverage, the 2D pair correlation function is no longer meaningful, as the molecules do not even approximately lie in a plane, as shown in the ESI.†

**Fig. 9:**
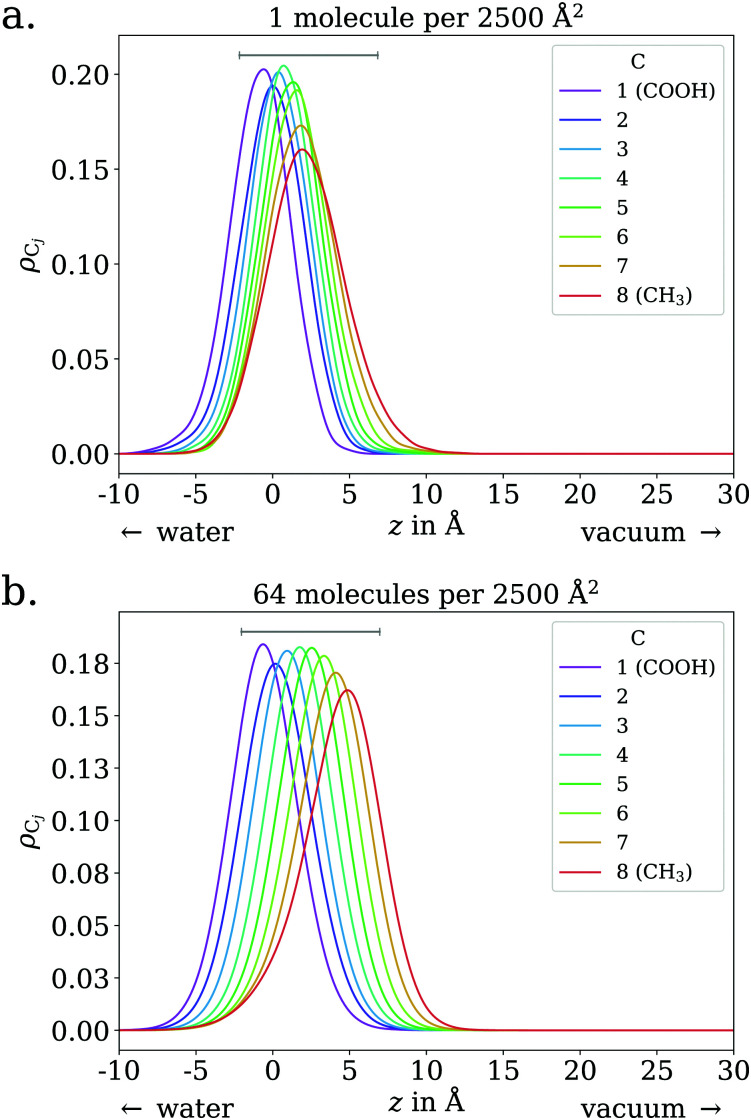
Vertical distributions of C atoms relative to the spatially averaged water surface at different coverages, for octanoic acid. Grey lines indicate 9 Å, the approximate distance between the carboxylic and methyl C atoms in one fully stretched-out octanoic acid molecule. The lower end of these lines is placed at the lower quartile of the carboxylic C atom's vertical distribution.

In the intermediate coverage regime (≲1 ML), the carboxylic groups of all acid molecules still adsorb on the water surface. This can be seen firstly from their relative vertical distribution (Fig. 6), which is remarkably indifferent to the coverage across both the low and ≲1 ML coverage range. Secondly, their 2D pair correlation function (Fig. 8a) still decays to 0 around *r* = 0, further confirming that the carboxylic groups are not layered above each other, but are all in contact with the aqueous phase. However, a clear trend can be observed here, in contrast to the low coverage regime. With increasing coverage, the pair correlation function becomes flatter as the water surface fills with acid molecules. While the molecules do tilt into a more upright position, compared to the low coverage limit, thermal motion still plays a big role and it does not appear like all molecules are vertically stretched out and horizontally aligned under any circumstances. This can be seen from the significant overlap of the vertical distributions of the different C atoms in Fig. 9b. The fact that it happens synchronously with the flattening of the pair correlation function, which indicates filling of the water surface, implies that the upward tilting of the alkyl chains happens mainly as a result of steric crowding.

At higher coverages, there is no longer enough space on the surface for all carboxylic groups to be in contact with water. These coverages were not covered in the experiments reported in the present work, and shall thus be addressed only briefly. The excess molecules adsorb on top of the surfactant layer, rather than moving into bulk water, as seen in the filling of the pair correlation function around *r* = 0 (Fig. 8a) and the vertical distributions of the C atoms, shown in the ESI.† This reflects the low solubility of octanoic acid in water at low pH, *cf.* Section 3.1. The exact structure of the octanoic acid layer is complex, and no definitive conclusions can be drawn from the two calculations performed in this range.

### 4.2 Anion

**Fig. 10:**
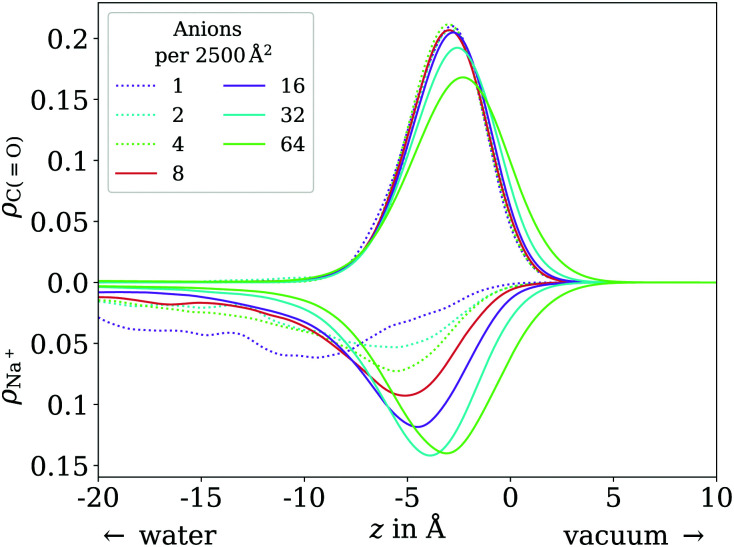
Vertical distribution of the carboxylic C atoms of octanoate (upward *y* axis) and Na^+^ counterions (downward *y* axis), in the low (dotted lines) and ≲1 ML coverage (solid lines) regimes.

In contrast to the acid, the anion has a considerable solubility in water.^54^ The actual surface coverages may therefore be smaller than simply the number of molecules in the simulation cell per cross section area. The fraction of molecules actually at the surface can be obtained by integrating over the peak near the surface in the vertical distribution of the carboxylic C atom. In practice, we find this integral to be exactly 1 for most of the trajectories up to 64 molecules per unit cell. Only for 8 molecules per unit cell we find on average 7.23 molecules at the surface and for 64 molecules per unit cell, we find on average 59.74 at the surface. These effective coverages were used for all further analyses, although we still use the total number of molecules per unit cell as labels in Fig. 7, 8 and 10 for the sake of simplicity. Only carboxylic C atoms in the range of −15 Å < *z* < 5 Å were considered in the 2D pair correlation functions.

Furthermore, sodium octanoate is a solid at room temperature.^54^ Accordingly, we observe the formation of an amorphous solid layer for 256 molecules per unit cell. This system does not equilibrate on the simulated timescale. Already at 128 molecules per unit cell, we observe aggregation of octanoate ions to some degree, rendering our analysis methods inappropriate. Thus, we further analyze only the simulations up to 1 ML coverage. For the sake of comparison between acid and anion, we assumed that the regimes described in the previous section are transferable from the observations made with the acid molecule.

In the low coverage range, the molecules are already more stretched out than they are in the acid, *cf.*Fig. 7. This can be attributed to the stronger solvatization of carboxylate compared to the neutral carboxylic group, resulting in a lower average vertical position of the carboxylic C atom. At the same time, the repulsion between the alkyl chain and bulk water is mostly unchanged, and the equilibrium between both effects leads to an orientation of the molecule where the carboxylate group is inside bulk water while most of the alkyl chain is on the water surface or in vacuum. Similar to the neutral acid, the vertical distribution of the carboxylic C atom does not vary across the low coverage range, as seen in Fig. 10. However, we observe accumulation of Na^+^ counterions near the surface with increasing coverage already in the low coverage range.

The trend of Na^+^ accumulating at and moving closer to the surface with increasing coverage is continued into the ≲1 ML coverage range, with the carboxylic C atoms now following that trend, albeit less pronounced. We conclude that the shift of the anion towards the surface at higher coverage is a result of the formation of a double layer. The vertical distributions of the carboxylic C atoms become wider rather than sharper with increasing coverage, indicating that thermal motion does play a role and complete alignment of the molecules never occurs, even for the anion. Similarly to the acid, the anions orient more along the *z* axis with increasing coverage. However, since the anions are more oriented than the acid molecules already in the low coverage range, this trend is much less pronounced for the anion, as seen in Fig. 7. In the 2D pair correlation (Fig. 8b) we observe the opposite trend as for the acid, with the first coordination shell rather than the long distance region filling up with increasing coverage. This can be attributed to repulsive Coulomb interactions between the negatively charged ions.

In addition to the results reported here, we also investigated the thickness of the surfactant layer and its coverage dependence, both for the acid and the anion. The observations largely match those made for the mean positions of the C atoms, shown in Fig. 7, and are thus reported only in the ESI† of this work.

## 5 Discussion

Before considering the PAD results, let us first discuss what information can or cannot be obtained from simple XPS signal intensities measured at a single detection angle. The interpretation of the surfactant layer structure at the liquid–vapor interface based on XPS data is complicated by the numerous factors that are contributing to the signal. The photoelectron peak intensities depend, among other factors, on the probability of inelastic scattering of the photoelectron from its point of emission to the detector. Although absolute signal intensities are difficult to use for quantitative analysis in XPS, relative signal intensities can be compared to each other to yield information on the relative position of atoms at the interface. For instance, in the present case the peak area ratio between the COO^−^ (COOH) and CH_*x*_ peaks depends on average only on the degree of inelastic scattering for photoelectrons from each of these species, since all other factors determining the photoelectron intensity are equal (*e.g.*, molecular density or transmission function of the electron analyzer) or can be corrected for using a gas-phase reference (photoionization cross-section). However, this ratio can be affected by several different factors related to the molecular arrangement at the water–vapor interface,^23^ such as:

• The position of the atoms relative to the interface, which affects the inelastic scattering probability by water molecules.

• The density of the surfactant layer, which affects the inelastic scattering probability by neighboring surfactant molecules.

• The orientation of the surfactant molecules at the interface.

The measured CH_*x*_ to COO (sum of COO^−^ and COOH) intensity ratio of the different solutions that were investigated is summarized in Table 1. This ratio is systematically higher than 7, *i.e.*, the expected stoichiometric ratio for octanoic acid, even after normalization to the gas-phase ratio, which already exhibited a 10% increase from the expected ratio. The ratio in solution increases with increasing bulk concentration. It is also higher for sodium octanoate compared to octanoic acid solutions. These observations are all consistent with several other previous XPS studies of surfactant solutions (alcohols, carboxylic acids, and amines),^19,20,53^ although in these other studies gas-phase ratios had not been measured.

The CH_*x*_ to COO (or any other terminal functional group) ratio has been suggested to be an indication of the orientation of the molecules at the interface. However, ambiguities remain since the ratio can also be affected by the other above-mentioned factors. For instance, it has been concluded that the increase of the CH_*x*_ to COO intensity ratio with bulk concentration, systematically studied in the case of alcohol surfactants,^8^ indicated a progressive change of orientation of the molecules going from relatively flat on the surface to standing up. This is fully backed up by MD simulations in the cited study. Similar conclusions could be drawn in the present study: our MD simulations confirm that octanoic acid, and to a lesser degree sodium octanoate, tend to be more aligned with the surface normal with increasing surface concentration, as one would intuitively expect. It would be tempting to connect this behaviour with the increase of the CH_*x*_ to COO ratio with increasing concentration. However, this ratio can increase due to a change of orientation of the molecules but also due to an increased surface concentration of molecules. The two effects cannot be disentangled here, and it is thus not possible to draw unambiguous conclusions based on this experimental data alone.

Some scenarios are even more unfavourable: in a solution that contains multiple species, such as in the case in Fig. 4b, it is impossible to obtain information on the relative depth below the surface of these species from the signal intensities since the density distribution of the species at the interface is not known a priori. Note that the CH_*x*_ to COO ratio is meaningless here as well. To get more information on the depth distribution, depth-profiling would have to be performed, with all the complications related to poorly constrained mean free paths, cross-sections, and so on. Comparison of the *β*′ values, on the other hand, can give a clear indication. This is precisely the type of information that can be extracted from the PADs measured in this work, even though, as we will now discuss, some caution is still required.

PADs depend on the elastic scattering probability. Indeed, elastic scattering changes electron trajectories and thus broadens the angular distribution, leading (for positive initial *β* values) to a reduction of *β*. The more elastic scattering is experienced by the photoelectrons, the more the distribution will tend towards isotropy (*β* = 0). Consequently, we measure lower values in the condensed phase than in the gas phase.

Elastic scattering is affected by the exact same factors listed above for inelastic scattering: the position of the atoms relative to the interface, the density of the surfactant layer, and the orientation of the surfactant molecules. The major difference is that values do not depend on other factors such as photoionization cross-section (although it does depend on photon energy, which therefore remains fixed in the present work) or atomic density, like in the case of XPS intensities.

In the observed *β*′ (normalized to the gas phase) values compiled in Table 1, the hierarchy is as follows: *β*′(CH_*x*_) > *β*′(COOH) > *β*′(COO^−^). One could interpret this as showing that the average position of the carbon of these different groups relative to the surface follows the same trend, with the COO^−^ group being deepest below the surface. However, caution is advisable when comparing results from different solutions. As explained above, the *β*′ values also depend on the surface density and arrangement of the surfactant molecules. Similar *β*′ values could be obtained from two systems where one has molecules deeper below the surface, but a lower surface density. In fact, the role of surface density is well demonstrated when we compare octanoic acid solutions and sodium octanoate solutions at different concentrations: we can see in Table 1 that the *β*′values all systematically increase when the bulk concentration (and thus also the surface density of surfactants) decreases. Scattering by the surfactant molecules therefore does play a role in the observed *β*′ values.

With this being established we can nonetheless draw a number of conclusions from the data presented above. For octanoic acid solutions, we observe *β*′(CH_*x*_) >*β*′(COOH), but as remarked earlier the two numbers are very close. For sodium octanoate solutions, the difference between *β*′(CH_*x*_) and *β*′(COO^−^) remains significant even after normalization. This confirms that on average the molecules are not lying flat on the surface, but that the octanoic acid molecules are not standing upright as much as the octanoate molecules. This is in agreement with the results of the MD simulations: for octanoic acid, the average position of the different carbons (projected onto the surface normal) are all very close to each other, especially at low coverage, while for octanoate they are much further apart at all coverages, indicating more upright molecules.

We cannot compare directly the results from octanoic acid solutions and sodium octanoate solution when it comes to the differences between *β*′(COOH) and *β*′(COO^−^). However, for the intermediate pH solutions where both species are present, scattering by surfactant molecules is the same for all species. Thus, it is possible to unambiguously conclude that indeed, COOH groups are on average closer to the surface than COO^−^ group, a fact which is also confirmed by the results of the MD simulations. This conclusion could not have been reached on the basis of the XPS signal intensities measured at a single angle.

## 6 Conclusion

We have performed a joint experimental and theoretical study of the behavior of a model surfactant molecule, octanoic acid, at the liquid–vapor interface. On the experimental side, we explored the use of photoelectron angular distributions as a means to characterize the orientation and relative position of surfactants with respect to the liquid–vapor interface. MD simulations supported the experimental findings of a decrease in the distance to the liquid vapor interface of the COOH group in octanoic acid compared to the COO^−^ group in octanoate.

One conclusion from this study is that caution is required when characterizing molecules at the liquid–vapor interface with XPS, including still rarely-used tools such as PADs or more established ones like peak intensity ratios and depth profiling by changing the detection angle or photoelectron kinetic energy. Gas-phase XPS spectra and PADs show differences in the anistropy and total cross-section of two very common carbon groups (CH_*x*_ and COOH), even at kinetic energies of 155 eV above the ionization threshold, which means that the measurement of gas-phase data – wherever possible – is a prerequisite to the proper analysis of condensed phase data. In addition, multiple factors enter into the degree of elastic and inelastic scattering experienced by photoelectrons of surfactant molecules at the solution–vapor interface, such as molecule orientation and the coverage. This complicates the comparison of XPS results for different solutions.

Using PADs it is nonetheless possible to obtain information on the relative position of surfactant molecules under conditions where angle-independent XPS does not allow any conclusion. Our complementary MD simulation and PAD measurements show that octanoate molecules are more upright than octanoic acid molecules at the interface, and that the COO^−^ groups are immersed deeper into the solution than the COOH groups. Although these conclusions follow intuition, they pave the way for more precise characterization of liquid–vapor interfaces with molecular-level photoemission tools and simulations.

## Author contributions

R. D. and J. F. wrote the manuscript with inputs from all authors. R. D. analyzed and interpreted the experimental data with input from H. B., B. W., and U. H. J. F. performed the MD simulations and interpreted the results with input from H. O. and K. R. R. S., C. N., and J. B. provided the beamline endstations and user support for the experiments. HB, BW, and R. D. conceived the experimental plans. R. D., C. R., F. T., T. B., C. N., U. H., B. W., and H. B. participated in the beamtimes where the data were acquired.

## Conflicts of interest

There are no conflicts to declare.

## Supplementary Material

CP-024-D1CP05621B-s001
