# Correction: Photoelectron angular distributions as sensitive probes of surfactant layer structure at the liquid–vapor interface

**DOI:** 10.1039/d3cp90161k

**Published:** 2023-08-09

**Authors:** Rémi Dupuy, Jakob Filser, Clemens Richter, Robert Seidel, Florian Trinter, Tillmann Buttersack, Christophe Nicolas, John Bozek, Uwe Hergenhahn, Harald Oberhofer, Bernd Winter, Karsten Reuter, Hendrik Bluhm

**Affiliations:** a Fritz-Haber-Institut der Max-Planck-Gesellschaft Faradayweg 4-6 14195 Berlin Germany Reuter@fhi-berlin.mpg.de Bluhm@fhi-berlin.mpg.de; b Chair for Theoretical Chemistry and Catalysis Research Center, Technische Universität München Lichtenbergstr. 4 85747 Garching Germany; c Helmholtz-Zentrum Berlin für Materialien und Energie Albert-Einstein-Str. 15 12489 Berlin Germany; d Institut für Kernphysik, Goethe-Universität Frankfurt am Main Max-von-Laue-Straße 1 60438 Frankfurt am Main Germany; e Synchrotron SOLEIL, L’Orme des Merisiers Saint-Aubin - BP 48, 91192 Gif-sur-Yvette Cedex France; f Chair for Theoretical Physics VII and Bavarian Center for Battery Technology, University of Bayreuth Universitätsstraße 30 95447 Bayreuth Germany

## Abstract

Correction for ‘Photoelectron angular distributions as sensitive probes of surfactant layer structure at the liquid–vapor interface’ by Rémi Dupuy *et al.*, *Phys. Chem. Chem. Phys.*, 2022, **24**, 4796–4808, https://doi.org/10.1039/D1CP05621B.

The authors would like to correct a typographical error on page 4798, right column, second paragraph of subsection 2.4, where the *ε* value of the Lennard-Jones potential used for the Na^+^ ions was reported with an incorrect unit as 0.01814 kcal mol^−1^. The correct unit is kJ mol^−1^. The full correct sentence should thus read:

“For sodium cations, we used a Lennard-Jones (LJ) potential with *ε* = 0.01814 kJ mol^−1^ and *σ* = 3.206 Å.”

The correct value of *ε* = 0.01814 kJ mol^−1^ was used in all calculations, and thus the results of the calculations and the conclusions based on them are not affected by this error.

The Royal Society of Chemistry apologises for these errors and any consequent inconvenience to authors and readers.

## Supplementary Material

