# Supplementary material for: Photoelectron angular distributions as sensitive probes of surfactant layer structure at the liquid–vapor interface
Source: Phys Chem Chem Phys. 2022 Feb 3;24(8):4796–808. doi: 10.1039/d1cp05621b (PMC8865751; doi:10.1039/d1cp05621b)
Supplement: CP-024-D1CP05621B-s001 [file CP-024-D1CP05621B-s001.pdf]

Electronic Supplementary Material (ESI) for Physical Chemistry Chemical Physics.
This journal is © the Owner Societies 2022

Electronic Supplementary Information to "Photoelectron angular distributions as sensitive probes of surfactant layer structure at the liquid-vapor interface"

Rémi Dupuy, Jakob Filser, Clemens Richter, Robert Seidel, Florian Trinter, Tillmann Buttersack, Christophe Nicolas, John Bozek, Uwe Hergenhahn, Harald Oberhofer, Bernd Winter, Karsten Reuter and Hendrik Bluhm

1 Model charges for MD simulations

Table 1: Charges generated by LigParGen[1, 2, 3], in elementary charges.

	**octanoic acid**	**octanoate**
C1-8	0.4395, −0.1908, −0.1673, −0.1752, −0.1747, −0.1762, −0.1757, −0.2361	0.4004, −0.2079, −0.1423, −0.1572, −0.1549, −0.1554, −0.1559, −0.2053
H(−C2-8)	0.1282, 0.1074, 0.0875, 0.0904, 0.0869, 0.0875, 0.0813	0.0692, 0.0774, 0.0651, 0.0729, 0.0715, 0.0738, 0.0647
O(=C)	−0.4704	−0.6375
O(−H)	−0.5487	N.A.
H(−O)	0.4560	N.A.

2 Uncorrected summary table

Table 2: Summary of the beta values and the ratio of CH_x to COO intensity (at the magic angle) for gas phase pentanoic acid and the different solutions. The values have NOT been normalized to gas phase values here.

	Pe. acid	Oc. acid (pH 3)		Oc. Acid/Octanoate		Na octanoate (pH 9)	
	Gas	4 mM	1 mM	4 mM pH 4.6	4 mM pH 4.9	15 mM	100 mM
Surf. dens. (molec/cm^2)		4 10^{14}	2.8 10^{14}			1.8 10^{14}	4 10^{14}
CH_x	1.96	1.64	1.69	1.68	1.69	1.63	1.60
COOH	1.87	1.54	1.57	1.56	1.57		
COO^-				1.47	1.49	1.43	1.41
CH_x/COO ratio	4.4	8.9	8.0	8.8	9.0	8.8	10.4

3 Additional MD data

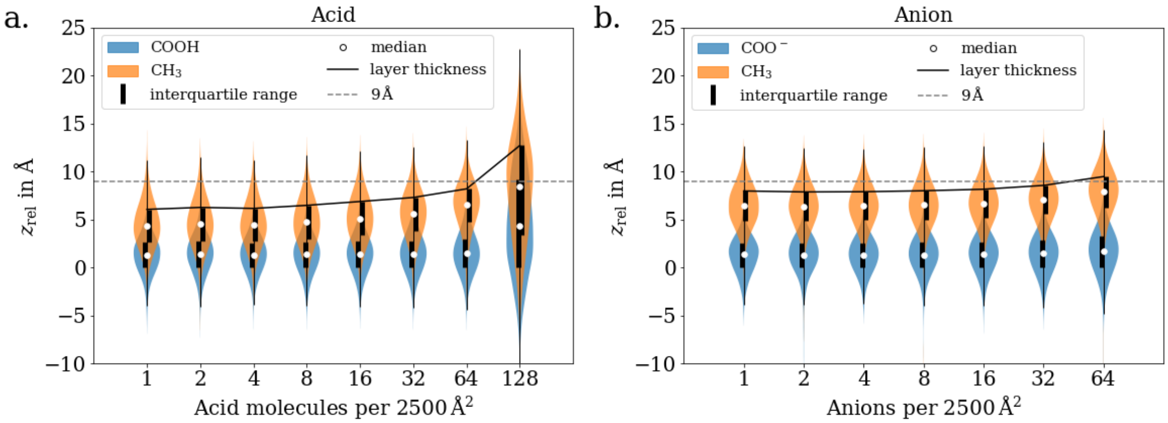

Figure S1: Violin plots of the carboxylic and methyl C atoms' vertical distributions, for octanoic acid (a.) and octanoate (b.). z_{rel} shifted such that the lower quartile for the carboxylic C atom is at zero. In a., these shifts range from -2.0 Å to -2.24 Å, with an average of -2.14 Å. In b., they range from -4.16 Å to -4.52 Å, with an average of -4.35 Å. Upper quartile of the methyl C atom used to estimate layer thickness. Approximate distance of 9 Å between the two C atoms for a fully straightened octanoic acid molecule shown for reference. For 128 molecules per unit cell, it was assumed that 1 ML is approximately $4 \cdot 10^{14}\,\mathrm{cm}^{-2}$, cf. sec. 3.1 in the main article. With $5.12 \cdot 10^{14}\,\mathrm{cm}^{-2}$, this simulation thus corresponds to ≈ 1.25 ML. In order to define the layer boundaries by the quartile of the first ML, we therefore used the 20% quantiles for 128 molecules per unit cell instead of quartiles. Data for 256 molecules per unit cell not shown, as the layer structure is too complex for this simplistic analysis, cf. fig. S2 b.

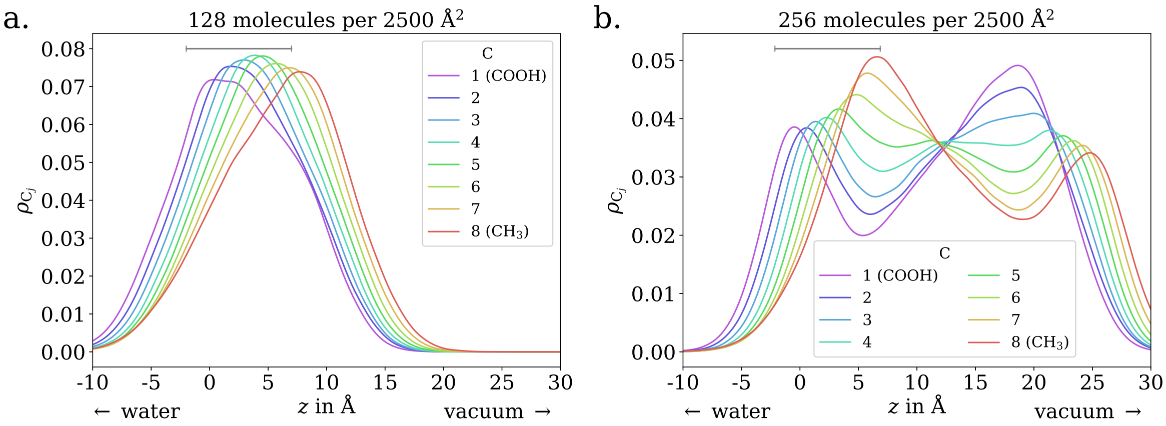

Figure S2: Vertical distributions of C atoms relative to the spatially averaged water surface at different coverages, for octanoic acid. Grey lines indicate 9 Å, the approximate distance between the carboxylic and methyl C atoms in one fully stretched out octanoic acid molecule. For 128 molecules per simulation cell (a.), the lower end of this line is placed at the shift from fig. S1, i.e. the 20% quantile of the carboxylic C distribution. For 256 molecules per cell (b.), such a quantile based analysis was not possible due to the complex layer structure. The average value of all z shifts from fig. S1 a. was used instead to place the 9 Å indicator.

References

[1] W. L. Jorgensen, J. Tirado-Rives & B. J. Berne; "Potential Energy Functions for Atomic-Level Simulations of Water and Organic and Biomolecular Systems"; Proceedings of the National Academy of Sciences of the United States of America **102**, pp. 6665–6670 (2005).

[2] L. S. Dodda, J. Z. Vilseck, J. Tirado-Rives & W. L. Jorgensen; "1.14*CM1A-LBCC: Localized Bond-Charge Corrected CM1A Charges for Condensed-Phase Simulations"; The Journal of Physical Chemistry B **121**, pp. 3864–3870 (2017).

[3] L. S. Dodda, I. Cabeza de Vaca, J. Tirado-Rives & W. L. Jorgensen; "LigParGen web server: an automatic OPLS-AA parameter generator for organic ligands"; Nucleic Acids Research **45**, pp. W331–W336 (2017).
